# COVID-19: how has a global pandemic changed manual therapy technique education in chiropractic programs around the world?

**DOI:** 10.1186/s12998-021-00364-7

**Published:** 2021-02-01

**Authors:** Katie de Luca, Marcus McDonald, Laura Montgomery, Stephen Sharp, Anika Young, Simon Vella, Michelle M. Holmes, Sasha Aspinall, Danica Brousseau, Chris Burrell, David Byfield, Dawn Dane, Philip Dewhurst, Aron Downie, Roger Engel, Brian Gleberzon, Dana Hollandsworth, Anne Molgaard Nielsen, Laura O’Connor, David Starmer, Michael Tunning, Paul Wanlass, Simon D French

**Affiliations:** 1 Department of Chiropractic, Macquarie University, Sydney, NSW Australia; 2 Discipline of Chiropractic, RMIT University, Melbourne, Australia; 3 School of Chiropractic, AECC University College, Bournemouth, UK; 4 College of Science, Health, Engineering and Education, Murdoch University, Perth, Australia; 5 Department of Chiropractic, Université du Québec à Trois-Rivieres, Trois-Rivières, Canada; 6 Welsh Institute of Chiropractic, University of South Wales, Pontypridd, UK; 7 Central Queensland University, Brisbane, Australia; 8 Undergraduate Education, Canadian Memorial Chiropractic College, Toronto, Canada; 9 Department of Chiropractic Sciences, Parker University, Dallas, USA; 10 Department of Sports Science and Clinical Biomechanics, University of Southern Denmark, Odense, Denmark; 11 Department of Chiropracti, Durban University of Technology, Durban, KwaZulu-Natal South Africa; 12 Associate Dean of Clinical Sciences, Palmer College of Chiropractic, Davenport, USA; 13 Interim Chair, Principles and Practice Department, Southern California University of Health Sciences, Los Angeles College of Chiropractic, Los Angeles, USA

**Keywords:** Chiropractic, Education, Manual therapy, Chiropractic technique, COVID-19, qQualitative.

## Abstract

**Background:**

Manual therapy is a cornerstone of chiropractic education, whereby students work towards a level of skill and expertise that is regarded as competent to work within the field of chiropractic. Due to the COVID-19 pandemic, chiropractic programs in every region around the world had to make rapid changes to the delivery of manual therapy technique education, however what those changes looked like was unknown.

**Aims:**

The aims of this study were to describe the immediate actions made by chiropractic programs to deliver education for manual therapy techniques and to summarise the experience of academics who teach manual therapy techniques during the initial outbreak of COVID-19 pandemic.

**Methods:**

A qualitative descriptive approach was used to describe the immediate actions made by chiropractic programs to deliver manual therapy technique education during the COVID-19 pandemic. Chiropractic programs were identified from the webpages of the Councils on Chiropractic Education International and the Council on Chiropractic Education – USA. Between May and June 2020, a convenience sample of academics who lead or teach in manual therapy technique in those programs were invited via email to participate in an online survey with open-ended questions. Responses were entered into the NVivo software program and analysed using a reflexive thematic analysis by a qualitative researcher independent to the data collection.

**Results:**

Data from 16 academics in 13 separate chiropractic programs revealed five, interconnected themes: Immediate response; Move to online delivery; Impact on learning and teaching; Additional challenges faced by educators; and Ongoing challenges post lockdown.

**Conclusion:**

This study used a qualitative descriptive approach to describe how some chiropractic programs immediately responded to the initial outbreak of the COVID-19 pandemic in their teaching of manual therapy techniques. Chiropractic programs around the world provided their students with rapid, innovative learning strategies, in an attempt to maintain high standards of chiropractic education; however, challenges included maintaining student engagement in an online teaching environment, psychomotor skills acquisition and staff workload.

**Supplementary Information:**

The online version contains supplementary material available at 10.1186/s12998-021-00364-7.

## Background

As a healthcare service, chiropractic care offers a conservative management approach with manual therapy at its foundation [1]. Registration or licensure into the profession is based on clinical competencies, which include the acquisition of relevant knowledge, understanding, attitudes, habits and psychomotor skills. The teaching of manual therapy is a cornerstone of chiropractic education, whereby students work towards a level of skill and expertise in manual therapy (emphasised by spinal manipulation) that is regarded as imperative within the field of chiropractic. To gain expertise in manual therapy, students must sequentially gain competency along a continuum of psychomotor complexity, which requires multiple semesters of instruction and practice [2, 3].

Manual therapy education has traditionally relied on face-to-face, hands-on teaching. For example, in the classroom setting, educators provide a theoretical, biomechanical description of the technique followed by a physical demonstration, after which the student engages psychomotor learning by practicing the technique themselves. Educators may provide real-time feedback to the students both verbally and by providing direct tactile modifications to students as they practice. Assessment of psychomotor skills is often completed via practical demonstration by students. One of the hallmarks for student readiness to practice in the chiropractic clinical setting is the ability to receive feedback on skills development and to be involved in work integrated learning [4, 5].

By March 2020, the COVID-19 pandemic placed many regions around the world into lockdown. Social distancing measures were enforced by state and federal governments in order to decrease the transmission rate of COVID-19 infection. In July 2020, measures included socially isolating and only leaving the home for essential activities such as employment, medical appointments, grocery shopping and physical activity. Physical distancing measures included keeping at least 1.5 m away from others, avoiding physical greetings such as handshakes, hugs and kisses, and minimising public transport and traveling at quieter times to avoid crowds.

Chiropractic programs around the world were faced with immediate and enforced changes to the intra (institutional) and inter (Faculty or school) academic environment. For example, the Council on Chiropractic Education – USA encouraged chiropractic education programs to be flexible and creative in finding possible solutions in the assessment of meta-competency outcomes [6]. They encouraged the incorporation of patient recordings and simulations, student demonstrations, clinical case studies, review of patient files and clinical rationale discussions to be an appropriate option to evaluate chiropractic students.^4^ In the educational setting for other professoins who also use manual therapies, in response to the COVID-19 pandemic there have been major shifts to online learning, development of new modes of student instruction and increased and enhanced teaching of manual therapy techniques to restore and maintain respiratory function [7]. While chiropractic programs internationally have had to make rapid changes to the delivery of manual therapy education, the extent and type of these changes are unknown. Therefore, the aims of this study were to describe the immediate changes made by chiropractic programs to deliver manual therapy technique education and to summarise the experience of academics who involved with manual therapy technique education during the COVID-19 global pandemic.

## Methods

### Design

A qualitative descriptive approach [8], via an online survey with open-ended questions, was used to describe the immediate actions made by chiropractic programs to deliver manual therapy technique education during the COVID-19 pandemic. Qualitative research is focused on the perceptions and experiences of a person [9], which has allowed us to describe changes to chiropractic programs from the viewpoint of academics who lead and teach manual therapy techniques in these programs. Reflexive thematic analysis was used based on the constructivist worldview (acknowledging the participants and the researchers have their own unique perspectives) and an inductive analysis (coding from the data rather than from the researcher’s analytic preconceptions) [10].

This report conforms with the COnsolidated criteria for REporting Qualitative studies (COREQ) checklist [11] and is provided as supplementary material 1. Advice was sought from the Macquarie University Faculty of Science and Engineering Human Research Ethics Committee for the requirements for formal ethical approval. Due to no foreseeable risk of harm or discomfort from the participants in this study design, and as each participant consented and contributed as an author, formal ethics approval was deemed not necessary, and was not sought.

### Participants

We used a convenience sample to collect data from participants. We identified international chiropractic programs from the webpages of the Councils on Chiropractic Education International and the Council on Chiropractic Education – USA. Academics who lead or teach manual therapy technique education were invited, via email, to provide comments on the immediate actions made in order to continue to deliver manual therapy technique education during the COVID-19 pandemic within their program. In May 2020, an email with two attachments (a letter of invitation explaining the study aims and outcomes of the research, and a word document with the survey) was sent to 26 academics, from 18 separate chiropractic programs. Snowball sampling was conducted if an academic recommended a colleague who was better placed to answer the survey. The survey was open for 1 month (until June 2020). A reminder email was sent after 2 weeks from the initial invitation to remind academics who had not already completed the survey. Those academics who did not respond to the letter of invitation and subsequent survey were not included in this study.

### Data collection

Academics first answered demographic questions and questions about their associated chiropractic program. In addition, five open-ended questions designed to capture the impact of changes made within their chiropractic program during the COVID-19 pandemic were asked (Table 1). These questions focussed on their experiences as an educator, their perspective as an observer of students, challenging factors and the greatest opportunities that will come from any changes made within their chiropractic program.

**Table 1:** Demographics and open-ended questions designed to capture the impact of changes made within chiropractic programs in order to deliver manual therapy content due to the COVID-19 pandemic

**Name and highest degree:**	
**Academic Institution:**	
**Year level/s of student learners you teach:**	
**1.** In your chiropractic program, how has the delivery of manual skills TUTORIALS* changed during the COVID-19 pandemic? If the delivery has changed relative to each year level, specify the year level/s. [250 word limit]	
**2.** In your chiropractic program, how has the non-practical (theoretical) component/s that supports manual skills tutorials changed during the COVID-19 pandemic? [250 word limit]	
**3.** From your observation as an educator, how have changes to manual skills tutorials during the COVID-19 pandemic impacted the student learner experience for (i) attendance and participation; (ii) engagement; and (iii) acquisition of psychomotor and cognitive skills, In your chiropractic program? [250 word limit]	
**4.** Please comment on: a) the most challenging factor/s for teaching manual skills during the COVID-19 pandemic; and b) what you see as the greatest opportunity that will come from the changes needed to teach manual skills during the COVID-19 pandemic? [250 word limit]	
**5.** Is there anything else you would like to include about how COVID-19 has impacted the teaching of manual therapy techniques in your chiropractic program? [100 word limit]	

### Qualitative analysis

Data were entered into the NVivo software program and coded and analysed by an experienced qualitative researcher independent to the data collection (MH). Thematic analysis was used according to Braun and Clarke [12]. Initial inductive coding was taken after reading all the responses, both describing and interpreting the content of the responses. Codes were examined and refined based on similarities and differences between codes. In this step, themes were generated based on clustering of codes to broad topics, which reviewed against the coded data, to explore if themes accurately depicted participants responses. Definitions of themes were created, and four authors met to discuss the generation of themes, ensuring themes had a clear focus, told a story of the data, and were relevant to the research question (KD, MM, MH, LM). All authors were responsible for the interpretation and discussion of the thematic analysis of the immediate actions made by chiropractic programs and the impact of these changes on the educator and (observed) learner experience.

## Results

**Fig. 1:**
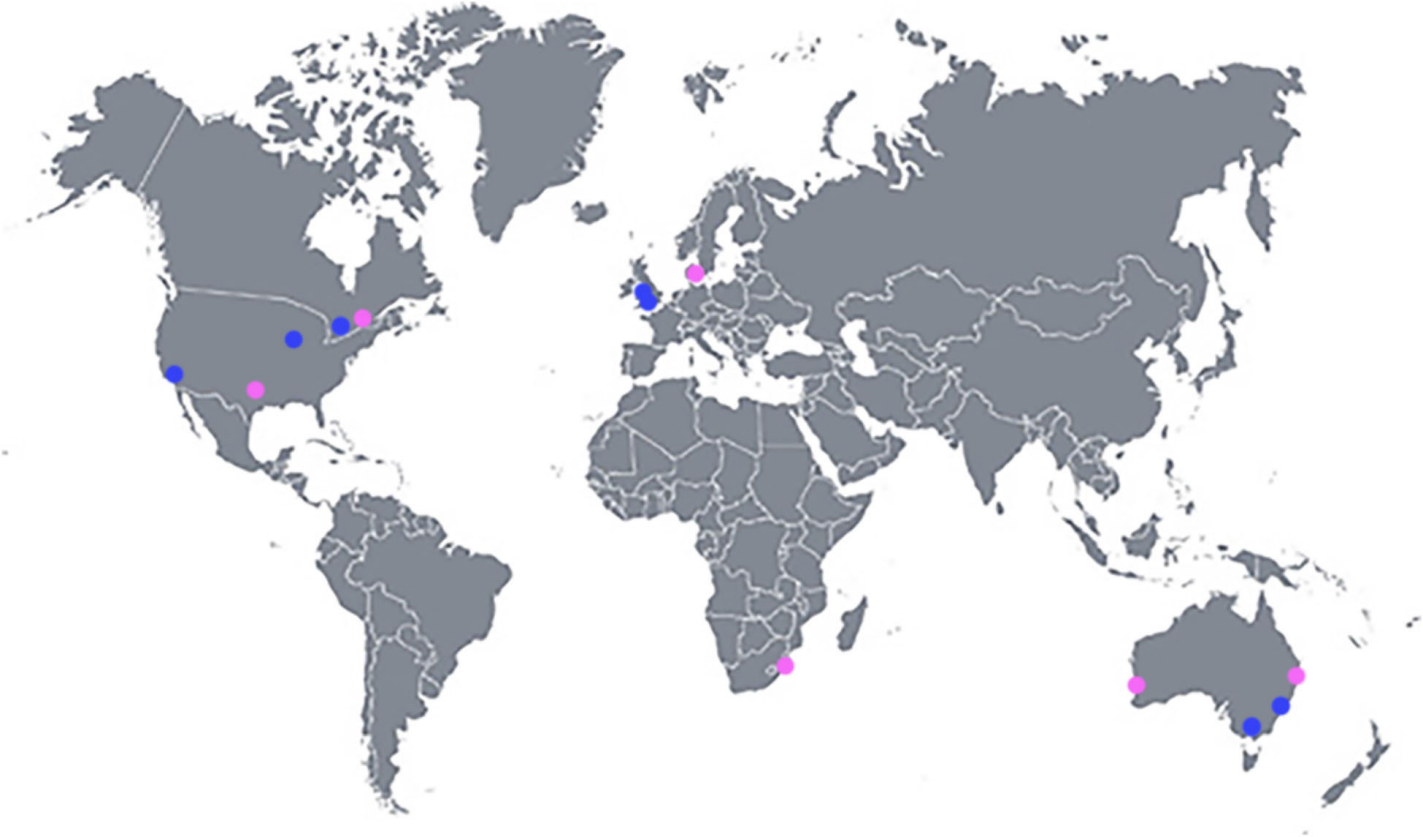
A visual description of the global geographical locations of the academics (females are pink dots and males blue dots), representing their respective programs

We received comments from 16 academics in 13 separate chiropractic programs (a 61.5% response rate from academics and a 72.2% response rate from chiropractic programs). Academics came from the following chiropractic programs (in alphabetical order): AECC University College, Canadian Memorial Chiropractic College (2), Central Queensland University, Durban University of Technology, Macquarie University (3), Murdoch University, Palmer College of Chiropractic, Parker University, RMIT, Southern California University of Health Sciences, Université du Québec à Trois-Rivières, the University of South Wales and the University of Southern Denmark. A visual description of the global geographical locations of the academics, and respective programs can be seen in Fig. 1.

Characteristics of the academics who responded are shown in Table 2. Most of the academics (12/16 (75%)) taught into multiple year levels, and one academic had no direct teaching load. In total, academics taught across all year levels. Year levels 1 to 3 had the equal and largest representation of respondents in this sample.

**Table 2:** Characteristics of the 16 academics who reported the immediate actions made in order to deliver manual therapy content due to the COVID-19 pandemic within their chiropractic program

Characteristic	Number (%)
Male	10/16 (63%)
Highest degree	
Bachelor of Chiropractic	1/16 (6%)
Master of Chiropractic	2/16 (13%)
Graduate Diploma of Chiropractic	1/16 (6%)
Doctor of Chiropractic	4/16 (25%)
Master of Health Science	2/16 (13%)
Master of Science	1/16 (6%)
Master of Research	1/16 (6%)
Master of Philosophy	2/16 (13%)
Doctor of Philosophy	2/16 (13%)
Academic Position^a^	
Associate Dean of Clinical sciences	1 (6%)
Head of department	3 (19%)
Professor	2 (13%)
Associate/assistant professor	3 (19%)
Senior lecturer/lecturer	7 (44%)
Education Coordinator	1 (6%)
Year of program taught into^a^	
Year 1	10
Year 2	10
Year 3	10
Year 4	6
Year 5	4
Doctorial	1
No direct teaching	1

^a^Academics were able to report teaching into multiple years of chiropractic programs and multiple academic positions, therefore a % could not be calculated, and only the accumulated number is presented

**Fig. 2:**
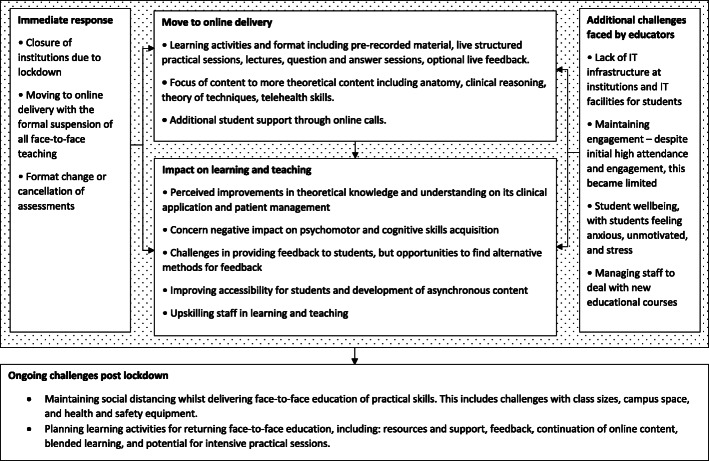
Five, inter-connected themes were generated by comments provided by academics whose chiropractic programs delivered manual therapy techniques during the COVID-19 pandemic

From the qualitative analysis, five interconnected themes were generated (Fig. 2) and are discussed below.

### An immediate response

Most notable were the government enforced, nation-wide public health orders that resulted in closures of institutions and campuses, rendering students and staff unable to attend their chiropractic program.

*The lock down was described in 5 levels, the first being total lockdown except for essential services. This resulted in the tertiary education institutions implementing an early recess as of the 18th March. … In terms of teaching manual therapies during the lock down period, there has been no direct tuition over the period of level 4 and 5 lockdowns. Instead this time has been utilised to move the university to a blended/multimodal approach for delivering the curriculum, starting 1st June* (Academic 13).

While individual durations of campus closures varied between countries, all programs had to formally suspend face-to-face teaching. The greatest immediate changes came in order to deliver manual therapy technique in small group, face-to-face, hands on (tutorial) sessions in an appropriate manner. Often, they were immediately ceased or delivered via *synchronous online meeting tutorials or asynchronous online videos to instruct students in exercises and techniques* (A11)*.* The immediate move to the online delivery of manual therapy technique education included changes in, or cancellations to, theory and psychomotor skills assessments. This included the addition of live video assessments, critiquing videos of technique performance, and one-to-one video vivas. Suspensions varied per program and guidance from national bodies was often sought for changes, for example, *All final written examinations for foundation, years 1–4 were converted to untimed online coursework submissions and cleared through Faculty QA procedures in consultation with the General Chiropractic Council (GCC) Education Committee for advice and support* (A4) while another academic explained, *Unfortunately, we have not been able to teach manual skills remotely during the pandemic. Our state board of chiropractic forbids students adjusting unless under the direct supervision of a licensed DC (A16).*

### Move to online delivery

The most significant change made by most chiropractic programs to deliver manual therapy technique education was the move to an online delivery of content, and that the focus of online content was theory driven. Academics developed creative solutions to the challenge, including live feedback of skills, live structured practice sessions, and clinical scenario workshops.

*All tutorials were conducted over Zoom with new material created or re-written …*

*delivered by creation of new videos to demonstrate both correct and incorrect versions of each procedure* (A7).

Changes to manual therapy technique content included covering aspects of anatomy, clinical reasoning and application, and tele-health skills via pre-recorded and live lectures, online question and answer sessions, and online discussion boards. Academics found frustration with the move to an online delivery, and it was felt that *there has been a significant drop in participation in online manual skills activities. Relatively few students have taken advantage of the feedback or practice sessions thus far* (A1). It was generally felt that academics are currently unable to grasp the full impact that changes will have on graduate skills. As mentioned, *the full extent of the effect on skills acquisition is not known as our academic year was near complete when the lockdown began, and we have not been able to observe students since* (A6).

### Additional challenges faced by academics

Challenges faced by academics were broad, and encompassed challenges at institutional, staff and student levels. Primarily, academics commented there was a general lack of information technology (IT) infrastructure at an institutional level, with many academics ill-prepared for the complete delivery of manual therapy techniques online, *COVID-19 has forced us to grapple with online technologies and re-visiting the status quo in terms of how we deliver the curriculum* (A13)*.* Additionally, stress from the urgency of delivering manual therapy technique education online (*rapid resource creation* (A3)) was palpable from academics, *there was no heads-up or preparation time to make changes to the teaching activities or to prepare new pedagogical material better adapted to distance learning* (A2), and that there should be a *very important distinction between emergency teaching methods and good online teaching practice. I feel most of what was done was laborious and yielded a less satisfactory learner and educator experience* (A11).

The majority of academics were senior lecturer or associate/assistant professors who oversee sessional staff employed to deliver manual therapy technique in tutorial sessions. Challenges faced by academics included *managing teaching staff who didn’t quite comprehend the change in education focus* (A3) and also those *with technical and resource challenges (computer literacy and online connectivity issues)* (A3)*.*

Most of the challenges faced by academics surrounded the (observed) learner experience. Understandably, comments included a lack of IT facilities for students and that online content was often being delivered remotely to the program. Specifically, *attendance varies due to a number of reasons including students not having a reliable internet connection or living in a different time zone* (A6)*.* The COVID-19 pandemic has resulted in a severe disruption to the student cohort, with previous classmates now residing in different countries. Chiropractic programs had to quickly enabled flexible, online education for uprooted students to *still participate in class through group assignments, message boards, direct email* (A15).

Maintaining engagement, at several levels, was evident as a challenge for academics. Despite initial high interest in online delivery, attendance at synchronous meetings and participation in tutorials waned over time. Online meeting platforms allowed for students to seem present but may indeed log in and have the session in the background or muted altogether. One respondent highlighted this experience by relating that *overall there is inconsistent attendance online. Some students log in but do not participate. Other students ask good questions online and participate online* (A10)*.* With another program echoing that it was evident that a small group of students were engaging and others hesitant to participate. *Typically, the same 4 or 5 students out of a group of 18 contributed* (A9). Aside from apparent interaction and participation, the respondents seem to commonly experience an actual attendance drop off.

*In the initial stage of the COVID-19 changes, attendance and participation was very high. Students readily took to the online mode of delivery. Apart from some early technical hurdles, this enthusiasm was maintained for approximately 4–6 weeks. However, around the 6-week mark, it became obvious that students began to become complacent with attendance and participation falling away noticeably in the weeks leading up to the end of semester assessments* (A8).

A narrative expressed throughout academic’s comments was the impact of the COVID-19 pandemic on students’ wellbeing, and the academics’ ability to manage student wellbeing. Academics reported student feelings of angst, anxiety, stress, unworthiness, disengagement and a lack of motivation to learn and a loss of confidence in their psychomotor skills: *This* [closure of institution] *resulted in a great deal of angst among students who are concerned they will be at a disadvantage compared to upper classmates* (A9). Academics noted that without *structure and specific timetabled psychomotor skills practical, some students will fail to engage as they will be somewhat disconnected from their cohort reducing peer to peer learning and practice sessions* (A4)*.*

### Impact on learning and teaching

With the focus of manual therapy technique content theory-driven, there were perceived improvements in theoretical knowledge and understanding on its clinical application and patient management.

*Student engagement related to theoretical components of psychomotor acquisition (delivery of force, adjustment mechanics, identifying errors in technique) potentially increased compared to previous offerings due to the greater volume and depth of content delivered in tutorials. Student learner experience related to acquisition of cognitive skills for assessment, diagnosis and management of basic spinal conditions has been superior to previous offerings* (A4).

Evidence that the immediate change to theory-driven content has enhanced the students’ cognitive ability was suggested, *cognitive skills seem to be developing well, possibly better than when in person, evidenced by the quality and depth of the questions that are asked* (A10). Academics shared that students have more thoroughly engaged in rich clinical discussions that otherwise would not have taken place, and there was a *deeper interaction within a theoretical framework may have enhanced student understanding of the role that manual therapy plays in overall patient management* (A7) and highlighted receiving positive student feedback from this approach.

Perhaps the most substantial impact of changes made within the chiropractic programs on students during the COVID-19 pandemic, and possibly at the expense of the improvements in theoretical knowledge, is the presumed negative impact on psychomotor skills acquisition. *Faculty have serious concerns about skill acquisition* (A10)*, psychomotor skill acquisition was severely hampered since there were no classes* (A3) and that academics had *no way to ensure safety by allowing students to practice psychomotor skills remotely* (A14), reveal the real concern that academics have for students’ psychomotor skill acquisition. Many programs will seek to mitigate any negative impact through the addition of more tutorial hours when classes reconvene or in the form of “CPD-styled” weekend workshops. The challenge and the uncertainty of teaching going forward was evident from several academics;

*The time for practising individual techniques are lacking. The content load will be massive in few days without having time for reflection and anchoring of the learned elements. It challenges the long-term memory* (A12).

*We currently do not know how we will recapture the lost time for students during the next academic year, scheduled to start at the end of August* (A9).

An important barrier to the learning of manual therapy techniques online that was highlighted by academics, was that *students at home do not have the correct equipment and are not insured to work on other people (A6).*Another impact on learning and teaching was the inability of academics to provide face-to-face feedback to students.

*The most challenging factor has been the lack of immediate feedback from students. It has been difficult to gauge the students’ progress without being able to easily and frequently observe their manual skills, which also makes it challenging to identify and address problem areas* (A1).

Academics provided comments that were on the whole were optimistic and positive. Many saw the value in the resources they had to rapidly create as useful in the future and indeed improving upon the past quality of teaching, *we have developed very useful educational resources that can continue to be used. This pandemic has also assisted in the uptraining of faculty in a lot of technology* (A14) and that going forward programs opportunities *will be to formulate and implement innovative methods of teaching psychomotor skills using high quality video presentations at all levels* (A4). Some educators extended their views of the response to the COVID-19 pandemic beyond their own program and suggested that with digital assets and online learning, perhaps there can be co-ordination of resources regionally and even internationally. *The access to recordings/videos of manual skills enhance the possibility to teach manual skills during a pandemic. This could lead to an opportunity of increased collaboration across different chiropractic programmes* (A12).

Additionally, the demand of moving to an online delivery provided a positive effect for several programs. Whereas previously the efforts of one person may have been enough to coordinate a given course or unit of study, the team effort that was necessary, involved “retooling” staff with an opportunity to upskill and increase their contribution. Academics expressed *surprising teaching stars rose to the challenges associated with online delivery. Excellent content creation from teachers that have otherwise not been involved in content creation* (A3). It was consistent among comments that it was the efforts of staff at a group level that made the pivoting and adaptation possible. Academics reported support came from a community of academic and teaching staff, rather than an institutional one.

#### Ongoing challenges and opportunities post lockdown

Academics were clear of the challenges that lay before them, particularly with the safe return of staff and students to campus, as the COVID-19 pandemic continues.

*I suspect there are many more new challenges to come. Right now, we have only dealt with delivering education remotely. Another likely challenge will be delivering our education in person but respecting potential social distancing requirements (max class sizes, minimum distances apart, max building/floor/room capacity, etc.)* (A14)*.*

*The return of face-to-face teaching of manual therapies in the time of COVID-19 brings a new dimension to the classroom. Social distancing measures, prevention of sharing of chiropractic tables and equipment, sanitising and ensuring the health and well-being of the staff and students will take incredible planning and self-monitoring. The lecturing staff will need to be dynamic and innovative to allow the acquisition of psychomotor skill in an environment that will at first, be quite foreign* (A13).

Also, of note, the reopening of chiropractic programs *will most likely occur under the provision that all students wear adequate personal protective equipment during all manual skills courses* (A2) and that *the use of perspex screens in small groups to permit face-to-face instruction will become the norm* (A2).

Ongoing challenging factors for academics teaching manual therapy techniques include the *definite limits to what we can teach and evaluate at a distance, without physically having direct contact and supervision* (A2) and ensuring competency of final year students to enter clinical practice. Specifically, *the most critical is for final year students who are concerned there will not be enough time for them to reach a sufficient level of competency expected of a new graduate and that this will impact their employment prospects* (A8).

## Discussion

The COVID-19 pandemic has caused immediate and substantial changes to the teaching of manual therapy techniques in chiropractic programs around the world. This study captured changes that were made, at one point in time, from May to June 2020. The COVID-19 pandemic is a rapidly evolving public health challenge, and at each of the chiropractic programs who participated in this research, different countries were in various stages of the COVID-19 pandemic. Also, countries responded in different ways, with inconsistencies in public health orders across countries. Some participants were only able to answer some of the questions due to being in complete lockdown (hence programs were closed), while others were in a position to reflect on the changes they had made as their countries were coming out of lockdown and resuming classes. Furthermore, heterogeneity in responses exist as programs were either at the end or start of their academic year. Cognisant of the diverse positions each chiropractic program was in, this independent qualitative analysis of comments from 16 academics in 13 separate international chiropractic programs has provided a rich, in depth summary of the experience of teaching manual therapy techniques during the initial COVID-19 outbreak. International chiropractic programs responded reactively in a rapidly changing environment, and key concepts of the immediate changes and experience from academics included the following.

**1. The cessation of face-to-face teaching, the move to online learning, and content was theory driven.** Many programs attempted to keep a synchronous delivery of content and used online collaborative workspaces or meeting-based electronic platforms. In support of these changes, it has been shown that the quality of learning (as measured by cognitive performance) of a synchronous online class was as equally effective to a physical classroom [13]. However, a permanent reduction in face-to-face tutorial sessions would likely impact student satisfaction as seen in other units of undergraduate education at a tertiary level, [14] and impact student psychomotor skill development, that hands on practice provides [15, 16]. It was presumed by academics that there will be a negative impact on the students’ psychomotor skills acquisition, however if and how these changes will influence the short, and long-term, psychomotor skills of their students is unknown. As the COVID-19 restrictions ease and students return to on-campus classes, additional tutorial time and/or re-sequencing of psychomotor training may be necessary for students to regain both confidence and competence in their ability to deliver manual therapy [3, 17].

**2. Challenges faced by academics were broad, and encompassed challenges at staff, student, and institutional levels.** Academic staff were required to immediately develop theory-based manual technique content with minimal preparation time to reduce disruption to the student learning experience. This led to a ‘snowballing’ effect where academics were required to re-develop formative assessment tasks to appropriately assess student competency based on understanding of the theoretical content behind manual techniques, instead of practical assessment of manual techniques. Furthermore, the reduced possibility of using face-to-face feedback on performance of manual skills (verbal and non-verbal) from teacher to students and among peers was a significant challenge, as this teaching and learning tool is known to be essential [18].

Student challenges that arose during the COVID-19 lockdowns were orientated more towards remote IT difficulties (i.e. internet issues, access to content) and the psychological impact associated with the loss of social interaction (i.e. maintaining motivation, loss of hands on group activities). The closure of sites meant students often lost access to IT infrastructure, which in some cases was the students only access to internet. Where possible, staff sought grants to assist students with internet access. The remaining IT issues were easily managed through trouble shooting. For example, secure links to online meetings were accessible via registration, and once students were registered, if their internet dropped out, they could easily rejoin the meeting. In addition, cameras/microphones whilst preferred, were not essential to engage with online discussions. Students were able to engage in the discussion through typing if they did not have access to a camera or microphone. It is suggested that research should be undertaken to survey the chiropractic student’s experience of online learning during the COVID-19 pandemic.

**3. The COVID-19 pandemic had, and is still having, a significant impact on staff and student wellbeing.** Social interaction is widely interlinked with psychological wellbeing and social opportunities and the restriction of these measures are suggested to be profoundly distressing to those experiencing strict isolation [19]. This study reveals, the lengths by which academics strived to maintain student engagement and to support their psychological well-being through regular online interaction and frequent email correspondence. In the return to teaching and future assessment of manual therapy technique education, chiropractic programs will need to take into account the mental impact of COVID-19 on staff and students. Chiropractic programs will need to actively promote student support resources (e.g. student wellbeing services, additional supervised classes and small-size group activities) to ensure that student mental health is monitored and appropriately managed. Additionally, in light of the intense and significantly increased workload by staff during the COVID-19 pandemic, and that staff workloads are currently augmented, programs should endeavor to implement strategies to manage staff wellbeing and maintain a productive workforce.

**4. There were a number of positives to emerge from this challenge.** Key changes that are likely to have a lasting impact on manual skills teaching, after the COVID-19 pandemic include the acquisition of technological know-how and the development of video learning resources to support manual skills learning online. High fidelity medical simulations have been successfully used to improve the psychomotor skill acquisition of doctors and nurses, [20–22] so, with the increased ability of staff to use technology, chiropractic programs should test and implement innovations in teaching manual therapy techniques that rely more on new technologies. Some academics expressed that students interacted with the online environment to a greater degree than if they were in face-to-face mode only. There may be opportunity to explore blended mode delivery in future offerings, though it is unclear what combination of online and face-to-face delivery will maximise student engagement.

Strengths of the study include the dependability of the data analysis, which was performed by an experienced, qualitative researcher (MH) who was independent to both the participant experience being surveyed (teaching manual therapy techniques in a chiropractic program) and the data collection process. While this could also be seen as a limitation of the study, themes were generated in consultation with three academics (KD, MM, LM) inherently involved with leading and teaching manual therapy technique during the COVID-19 pandemic. The use of NVivo allowed transparency of coding decisions and queries to confirm themes were coherent and meaningful to academics. This discussion and collaboration ensured the themes were sensitive to context. Our data analysis benefited from the proportion of academics from programs who answered the survey (13/18 (72%)), providing a depth to the investigation that enabled us to explore the impact of changes, challenging factors and greatest opportunities that will come from any changes made from chiropractic programs around the world.

There are limitations to our study. Results should be interpreted with caution due to the nature of convenience sampling as the population may not be generalizable to academics in other chiropractic programs, or in other disciplines. Academics were invited to be an author at the time of recruitment, which may introduce respondent bias whereby authors may contribute differently (whether more positively or negatively) than if they were participants alone. Furthermore, responses of the academics could also be influenced by the fact that they are reporting on changes at chiropractic programs they are employed by, where conflicts of interests may lie. Ultimately, we do believe that while participants were invited as authors, the information provided has not become less useful.

## Conclusion

This study used a qualitative descriptive approach to describe how some chiropractic programs responded to the COVID-19 pandemic in their teaching of manual therapy techniques.

This study was performed between May and June 2020, when national public health orders were first implemented due to the COVID-19 pandemic. This study used a qualitative descriptive approach to describe how some chiropractic programs immediately responded to the initial outbreak of the COVID-19 pandemic in their teaching of manual therapy techniques. Chiropractic programs around the world provided their students with rapid, innovative learning strategies, in an attempt to maintain high standards of chiropractic education; however, challenges included maintaining student engagement in an online teaching environment, psychomotor skills acquisition and staff workload.

## Supplementary Information

**Additional file 1.**

## Data Availability

The datasets used and/or analysed during the current study are available from the corresponding author on reasonable request.
