# Supplementary material for: COVID-19: how has a global pandemic changed manual therapy technique education in chiropractic programs around the world?
Source: Chiropr Man Therap. 2021 Feb 1;29:7. doi: 10.1186/s12998-021-00364-7 (PMC7849220; doi:10.1186/s12998-021-00364-7)
Supplement: Supplementary file 1 — Additional file 1. [file 12998_2021_364_MOESM1_ESM.docx]

**Supplementary Material 1 – Consolidated criteria for reporting qualitative studies (COREQ) checklist.**

| **Number** | **Item** | **Guide questions/description** |
| --- | --- | --- |
| **Domain 1: Research team and reflexivity** | | |
| **Personal Characteristics** | | |
| 1 | Interviewer/facilitator: | An invitation to participate in an online survey was delivered via email to potential participants from the lead author (KD). There was minimal interaction between the lead author and potential participants. |
| 2 | Credentials: | Credentials (highest degree) of the researchers are: Katie de Luca, PhD; Marcus McDonald, B Chiro Sci; Laura Montgomery, MRes, MChiro; Stephen Sharp MRes, MChiro; Anika Young, MRes, MChiro; Simon Vella, MChiro; Michelle M Holmes, MRes; Sasha Aspinall, PhD; Danica Brousseau, DC, MSc; Chris Burrell; David Byfield, MPhil; Dawn Dane, MSc (Chiro); Philip Dewhurst, MSc; Aron Downie, PhD; Roger Engel, PhD; Brian Gleberzon, DC; Dana Hollandsworth, DC; Anne Mølgaard Nielsen, PhD; Laura O’Connor, M. Tech Chiropractic; David Starmer, MHS; Michael Tunning, DC, ATC, MS; Paul Wanlass, DC; Simon French, PhD |
| 3 | Occupation: | All authors are employed within chiropractic programs identified from the webpages of the Councils on Chiropractic Education International and the Council on Chiropractic Education – USA. |
| 4 | Gender: | The lead author and facilitator of the survey is female (KD). The research team includes members who identify as both female and male genders. |
| 5 | Experience and training: | The lead author has a PhD in Medicine and Public Health from the University of Newcastle (Australia) (KD) and the qualitative researcher is a PhD student in Psychology from the University of Southampton, UK. Other experience and training is described above (credentials). |
| **Relationship with participants** | | |
| 6 | Relationship established: | The lead researcher (KD) and research team members (LM, SS, SV, AY, SF) work within the same chiropractic program as three participants (CB, AD, RE). A respondent to the survey and author (PD) works within the same program as the qualitative researchers (MH). |
| 7 | Participant knowledge of the  Interviewer: | Participants were introduced to the interviewer by an email, which contained a letter of introduction that included the aim of this project, details of the interviewer and that written responses to a survey would be qualitatively analysed to generate themes. |
| 8 | Interviewer characteristics: | Participants were told that the journal *Chiropractic & Manual Therapies* had called for articles relating to the chiropractic profession and the COVID-19 pandemic. The research team had intentions to submit a study documenting how the COVID-19 global pandemic has changed the teaching of manual therapy techniques across international chiropractic programs. |
| **Domain 2: Study design** | | |
| **Theoretical framework** | | |
| 9 | Methodological orientation and Theory: | A constructivist qualitative approach was taken to collect data and generate themes to understand the experience and perspective of academics teaching manual therapy techniques during the COVID-19 pandemic. |
| **Participant selection** | | |
| 10 | Sampling: | A convenience sample was taken of academics who lead or teach into manual therapy technique within Chiropractic programs worldwide. Programs were identified from the webpages of the Councils on Chiropractic Education International and the Council on Chiropractic Education – USA. |
| 11 | Method of approach: | An online, email invitation to participate was sent to potential participants. A reminder invitation was sent at two weeks if the potential participant had not responded. |
| 12 | Sample size: | 26 academics from 18 chiropractic programs were invited to participate. 16 academics from 13 chiropractic programs returned a complete survey. |
| 13 | Non-participation: | Multiple academics were invited from the same institution, and in three instances one academic wrote on behalf of program colleagues (SA, DB, BG, LO and DS). Academics from five programs did not respond (Northwestern Health Sciences University Minnesota, USA; New Zealand College of Chiropractic, New Zealand; University of Zurich, Switzerland; Institut Franco-Européen de Chiropraxie, France; Logan University, USA). No academic explicitly declined to participate. |
| **Setting** | | |
| 14 | Setting of data collection: | An online survey. |
| 15 | Presence of non-participants: | N/A |
| 16 | Description of sample: | See Table 2. |
| **Data** **collection** | | |
| 17 | Interview guide: | See Table 1 for survey questions. A draft survey was developed by the research team (KD, LM, SS, SV, AY, SF). The final survey was not pilot tested. Participants had a 250 word limit to answer the open-ended questions. |
| 18 | Repeat interviews: | Participants answered the survey once and no follow up questions were asked. |
| 19 | Audio/visual recording: | N/A |
| 20 | Field notes: | N/A |
| 21 | Duration: | The survey took approximately 15 minutes to complete. |
| 22 | Data saturation: Was data saturation discussed? | N/A |
| 23 | Transcripts returned: | Participants answered the survey questions independently and typed their own answers. Surveys were not returned to participants for reflective comment. |
| **Domain 3: analysis and findings** | | |
| **Data analysis** | | |
| 24 | Number of data coders: How many data coders coded the data? | Four authors (LM, SS, SV, AY) independently compiled and cross-checked data between researchers. One author (MH) entered and coded the data in NVivo. Four authors (KD, MM, MH, LM) discussed the generation of five themes. |
| 25 | Description of the coding tree: Did authors provide a description of the coding tree? | 74 initial codes were developed into 17 higher level codes. Higher level codes and themes are depicted in Figure 2. |
| 26 | Derivation of themes: | Themes were inductively derived from the data. |
| 27 | Software: | NVivo |
| 28 | Participant checking: | All participants had the opportunity to review the generated themes, give feedback and provided discussion of the thematic analysis of data. |
| **Reporting** | | |
| 29 | Quotations presented: | Quotations have been presented in the manuscript to illustrate themes, with a transcript ID provided in the narrative text. |
| 30 | Data and findings consistent: | Yes, there is consistency between the data collected, the themes generated and the discussion. Supplementary file 1 also provides evidence of consistency between the data and reported findings. |
| 31 | Clarity of major themes: | Yes, please see Figure 2 where five major themes are presented clearly; 1. Immediate response, 2. Move to online delivery, 3. Additional challenges faced by educators, 4. Impact on learning and teaching, and 5. Ongoing challenges post lockdown. |
| 32 | Clarity of minor themes: | Within each of the five major themes there are one to three minor themes presented in Figure 2, the results and discussion sections. |
